# Combining cytogenetic and epigenetic approaches in chronic lymphocytic leukemia improves prognosis prediction for patients with isolated 13q deletion

**DOI:** 10.1186/s13148-017-0422-7

**Published:** 2017-11-28

**Authors:** Cristina Bagacean, Christelle Le Dantec, Christian Berthou, Adrian Tempescul, Hussam Saad, Anne Bordron, Mihnea Zdrenghea, Victor Cristea, Nathalie Douet-Guilbert, Yves Renaudineau

**Affiliations:** 1 U1227 B lymphocytes and autoimmunity, University of Brest, INSERM, IBSAM, Labex IGO, networks IC-CGO and REpiCGO from “Canceropole Grand Ouest”, Brest, France; 2 Laboratory of Immunology and Immunotherapy, Brest University Medical School Hospital, BP 824, 29609 Brest, France; 3 “Iuliu Hatieganu” University of Medicine and Pharmacy, Cluj-Napoca, Romania; 4 Department of Hematology, Brest University Medical School Hospital, Brest, France; 5 Department of Hematology, “Ion Chiricuta” Oncology Institute, Cluj-Napoca, Romania; 6 Laboratory of Cytogenetics, Brest University Medical School Hospital, Brest, France

**Keywords:** Chronic lymphocytic leukemia, Cytogenetics, DNA methylation, Active DNA demethylation, DNMT, TET

## Abstract

**Background:**

Both defective DNA methylation and active DNA demethylation processes are emerging as important risk factors in chronic lymphocytic leukemia (CLL). However, associations between 5-cytosine epigenetic markers and the most frequent chromosomal abnormalities detected in CLL remain to be established.

**Methods:**

CLL patients were retrospectively classified into a cytogenetic low-risk group (isolated 13q deletion), an intermediate-risk group (normal karyotype or trisomy 12), and a high-risk group (11q deletion, 17p deletion, or complex karyotype [≥ 3 breakpoints]). The two 5-cytosine derivatives, 5-methylcytosine (5-mCyt) and 5-hydroxymethylcytosine (5-hmCyt), were tested by ELISA (*n* = 60), while real-time quantitative PCR was used for determining transcriptional expression levels of *DNMT* and *TET* (*n* = 24).

**Results:**

By using global DNA methylation/demethylation levels, in the low-risk disease group, two subgroups with significantly different clinical outcomes have been identified (median treatment-free survival [TFS] 45 versus > 120 months for 5-mCyt, *p* = 0.0008, and 63 versus > 120 months for 5-hmCyt, *p* = 0.04). A defective 5-mCyt status was further associated with a higher percentage of 13q deleted nuclei (> 80%), thus suggesting an acquired process. When considering the cytogenetic intermediate/high-risk disease groups, an association of 5-mCyt status with lymphocytosis (*p* = 0.0008) and the lymphocyte doubling time (*p* = 0.04) but not with TFS was observed, as well as a reduction of *DNMT3A*, *TET1*, and *TET2* transcripts.

**Conclusions:**

Combining cytogenetic studies with 5-mCyt assessment adds accuracy to CLL patients’ prognoses and particularly for those with 13q deletion as a sole cytogenetic abnormality.

## Introduction

Clinical heterogeneity is the hallmark of chronic lymphocytic leukemia (CLL), with some patients surviving for decades without needing treatment, while others present early progression and a short overall survival, if untreated [1]. Therefore, great efforts have been made in order to best predict CLL evolution at the time of diagnosis, and for this, the most widely used markers are cytogenetic parameters [2]. To this end, Dohner’s prognostic algorithm for clinical aggressiveness was proposed by combining conventional chromosome banding with the four main cytogenetic alterations detected by fluorescence in situ hybridization (FISH) since these abnormalities are present in more than 80% of CLL cases [3]. Indeed, patients with deletion (del)17p, del(11q), and a complex karyotype (≥ 3 chromosomal abnormalities) most frequently have an unfavorable outcome (high risk) followed by patients with trisomy 12 or a normal karyotype (intermediate risk). In contrast, over 30% of the cases are represented by patients with an isolated del(13q) and exhibit a favorable evolution (low risk). The main limitation of this cytogenetic approach is related to the fact that cytogenetic abnormalities can evolve during the course of the disease with new subclones which can substitute for those previously established, by presenting either new deletions (e.g., del(17p) or del(11q)) or driver mutations (e.g., *TP53*, *SF3B1*), and such an effect can be enhanced by chemotherapy [4].

In parallel, studies have investigated the outcome of CLL patients depending on epigenetic modifications as determined by epigenome-wide association studies (EWAS), genome-wide chromatin accessibility maps, and microRNA analysis showing that loss of epigenetic stability is associated with disease progression [5, 6]. The link between DNA methylation loss in promoters and a higher probability of harboring a subclonal driver mutation was highlighted, the consequence being an adverse clinical outcome [5]. The list of tumor regulator genes and markers with high prognostic significance, like *TCL1*, *CD20*, and *LPL*, which display altered DNA methylation regulation, is continuously growing [7–9]. DNA demethylation also occurs at repetitive elements, suggesting that global DNA demethylation reported in CLL largely accounts for repetitive elements that represent half of the chromatin [10, 11]. The mechanisms underlying aberrant DNA methylation in CLL are complex and do not rely only on the control of DNA methylation by DNA methyl transferases (DNMT). DNA demethylation, catalyzed by the ten-eleven translocation (TET) enzymes, has also been shown to be altered in CLL [12, 13].

The goal of the present work was to ascertain the associations between the main cytogenetic risk factors of the disease and the two cytosine derivatives: 5-methylcytosine (5-mCyt) and 5-hydroxymethylcytosine (5-hmCyt). *DNMT* and *TET* transcript levels were also studied by real-time quantitative (RTq)-PCR. The main observation highlighted by this study is that combining a conventional cytogenetic approach with global 5-mCyt assessment drastically improves CLL outcome prediction in patients with del(13q).

## Results

### Population characteristics according to cytogenetic risk groups

**Fig. 1:**
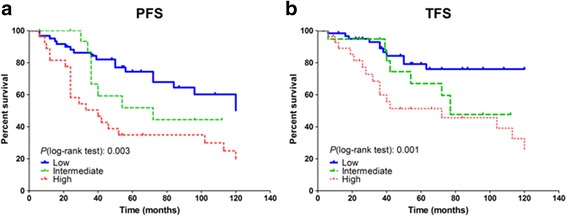
**a**, **b** Progression (PFS)- and treatment-free survival (TFS) according to cytogenetic risk groups. Kaplan–Meier survival curves from the date of diagnosis depicted for 127 chronic lymphocytic leukemia patients. Statistical differences between the curves were calculated using the log-rank test

Conventional chromosome and FISH analyses were performed, prior to treatment initiation, on the 127 CLL patients retrospectively included in the study at Brest University Hospital. Among them, 110 (86.6%) exhibited del(13q) which was isolated in 64 (50%) patients, 8 (6.3%) presented a normal karyotype, 11 (8.7%) had trisomy 12, 13 (10.2%) had a del(11q), 12 (9.4%) had a del(17p), and 15 (11.8%) had a complex karyotype with 3 or more cytogenetic abnormalities. Patients were segregated into cytogenetic risk groups according to the following classification: 64 (50%) patients with isolated del(13q) were in the low-risk group, 19 (15%) with normal karyotype or trisomy 12 were in the intermediate-risk group, and 30 (23.6%) with del(11q), del(17p), or complex karyotype were in the high-risk group. To be consistent with the cytogenetic classification criteria, 14 patients (11%) presenting 1–2 cytogenetic abnormalities, other than those aforementioned, were excluded from further analysis [2, 14]. In order to validate the cohort, we next evaluated the CLL outcome from the time of diagnosis, and as anticipated, the low-risk group had the longest median time to progression (PFS) and treatment initiation (TFS) (both > 120 months), followed by the intermediate-risk group (72 and 77 months, respectively) and the high-risk group (40 and 72 months, *p* = 0.003 and *p* = 0.001, respectively) (Fig*.* 1a, b). We further evaluated the association between the cytogenetic classification and prognostic factors such as age at diagnosis, sex, Binet stage, lymphocytosis, lymphocyte doubling time (LDT), immunoglobulin heavy chain variable region (*IGHV*) mutational status, and CD38 positivity (> 30%) (Table 1). Among these factors, lymphocytosis significantly progressed from the low-risk group to the high-risk group, and the highest levels were observed in the intermediate-risk group (*p* = 0.006). Additionally, median LDT was progressively shorter from the low-risk group to the high-risk group (*p* < 0.001), confirming that LDT, rather than lymphocytosis, better predicted disease progression.

**Table 1:** Patients’ characteristics according to cytogenetic risk groups

Cytogenetic risk groups	Low (*n* = 64)	Intermediate (*n* = 19)	High (*n* = 30)	Statistics (*p*)
Age at diagnosis, mean (±SD)	64.4 (± 9.4)	67.8 (± 8.2)	64.9 (± 10.7)	NS
Age at study entry, mean (±SD)	68.7 (± 9.3)	72.2 (± 8.4)	69.7 (± 9.8)	NS
Binet stage, No. of patients (%)				0.05
A	50/64 (78.1%)	11/19 (57.9%)	16/30 (51.7%)	
B	8/64 (12.5%)	5/19 (26.3%)	11/30 (37.9%)	
C	6/64 (9.4%)	3/19 (15.8%)	3/30 (10.3%)	
Lymphocytosis (Giga/L), mean (±SD)	30.7 (± 29.3)	74.7 (± 71.1)	49.6 (± 40.60)	0.006
IGHV mutational status, No. of patients (%)				NS
Unmutated (≥ 98% homology)	1/22 (4.5%)	2/12 (16.7%)	3/10 (30%)	
Mutated (< 98% homology)	21/22 (95.5%)	10/12 (83.3%)	7/10 (70%)	
CD38 > 30%, No. of patients (%)	12/58 (20.7%)	9/19 (47.4%)	6/25 (24%)	NS
LDT from diagnosis, median (months)^a^	48	24	17	0.0004
PFS, median (months)^a^	> 120	72	40	0.003
TFS, median (months)^a^	> 120	77	72	0.001

Abbreviations: *NS* not significant, *No.* number, *SD* standard deviation, *IGHV* immunoglobulin heavy-chain variable region, *LDT* lymphocyte doubling time, *PFS* progression-free survival, *TFS* treatment-free survival

^a^Kaplan–Meier survival analysis

### DNA methylation/hydroxymethylation and cytogenetic abnormalities

**Fig. 2:**
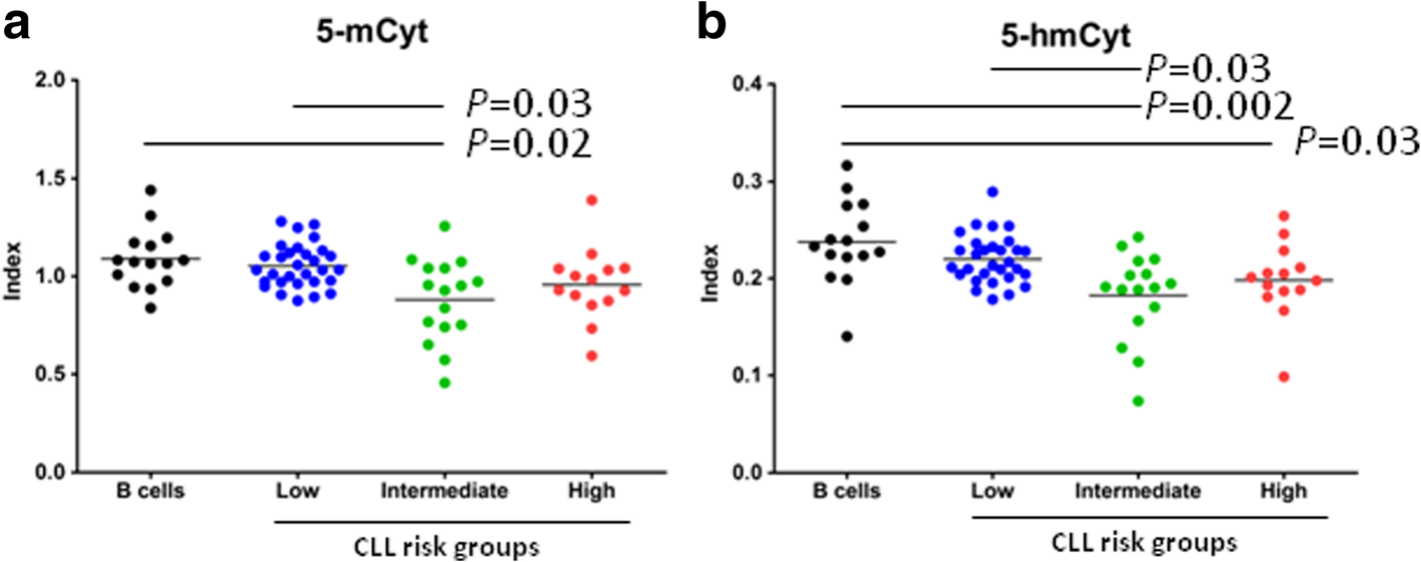
Global DNA methylation and hydroxymethylation according to cytogenetic risk groups **a** 5-methylcytosine (5-mCyt) and **b** 5-hydroxymethylcytosine (5-hmCyt) were determined by ELISA in purified B cells from 15 healthy controls and 60 untreated chronic lymphocytic leukemia (CLL) patients classified according to their cytogenetic risk group. ELISA results are expressed as indexes using a DNA reference sample for normalization (see “Materials and methods” section). The means and statistical differences were indicated when Dunn’s correction test was statistically significant (*p* < 0.05)

Next, for a comprehensive evaluation of DNA methylation/hydroxymethylation in CLL B cells and their interplay with the cytogenetic status, global 5-mCyt (Fig. 2a) and 5-hmCyt (Fig. 2b) were quantified by ELISA in purified peripheral blood B cells from 60 CLL patients randomly selected from the initial cohort and 15 healthy controls. The methylation status of the low-risk group (*n* = 30; 5-mCyt mean index 1.05 ± 0.02) and the high-risk group (*n* = 14; index 0.96 ± 0.05) were similar to healthy controls (index 1.09 ± 0.04, NS both), while a significant reduction was reported in the intermediate-risk group (*n* = 16, index 0.88 ± 0.05, *p* = 0.02). Regarding the hydroxymethylation status, levels of the low-risk group were similar to healthy controls (5-hmCyt mean index 0.22 ± 0.005 versus 0.24 ± 0.01, NS), while significant differences were observed for the intermediate-risk group (index 0.18 ± 0.04; *p* = 0.002) and the high-risk group (index 0.19 ± 0.03; *p* = 0.03).

### Bivariate analysis of cytogenetic and epigenetic characteristics

In order to further test the interplay between cytogenetic risk groups and epigenetic factors, a Cox regression analysis was performed on TFS for 5-mCyt and 5-hmCyt levels to segregate the 60 CLL patients tested by ELISA into subgroups with low and high levels. Next, two groups of patients were considered: first, the low-risk group with isolated del(13q) (*n* = 30) and, second, the cytogenetic intermediate/high-risk groups (*n* = 30). Concerning the latter, results were combined for statistical purposes, as the number of patients belonging to these two cytogenetic risk groups and classified as high for 5-mCyt or 5-hmCyt levels, was reduced (*n* = 5 and 7, respectively).

**Fig. 3:**
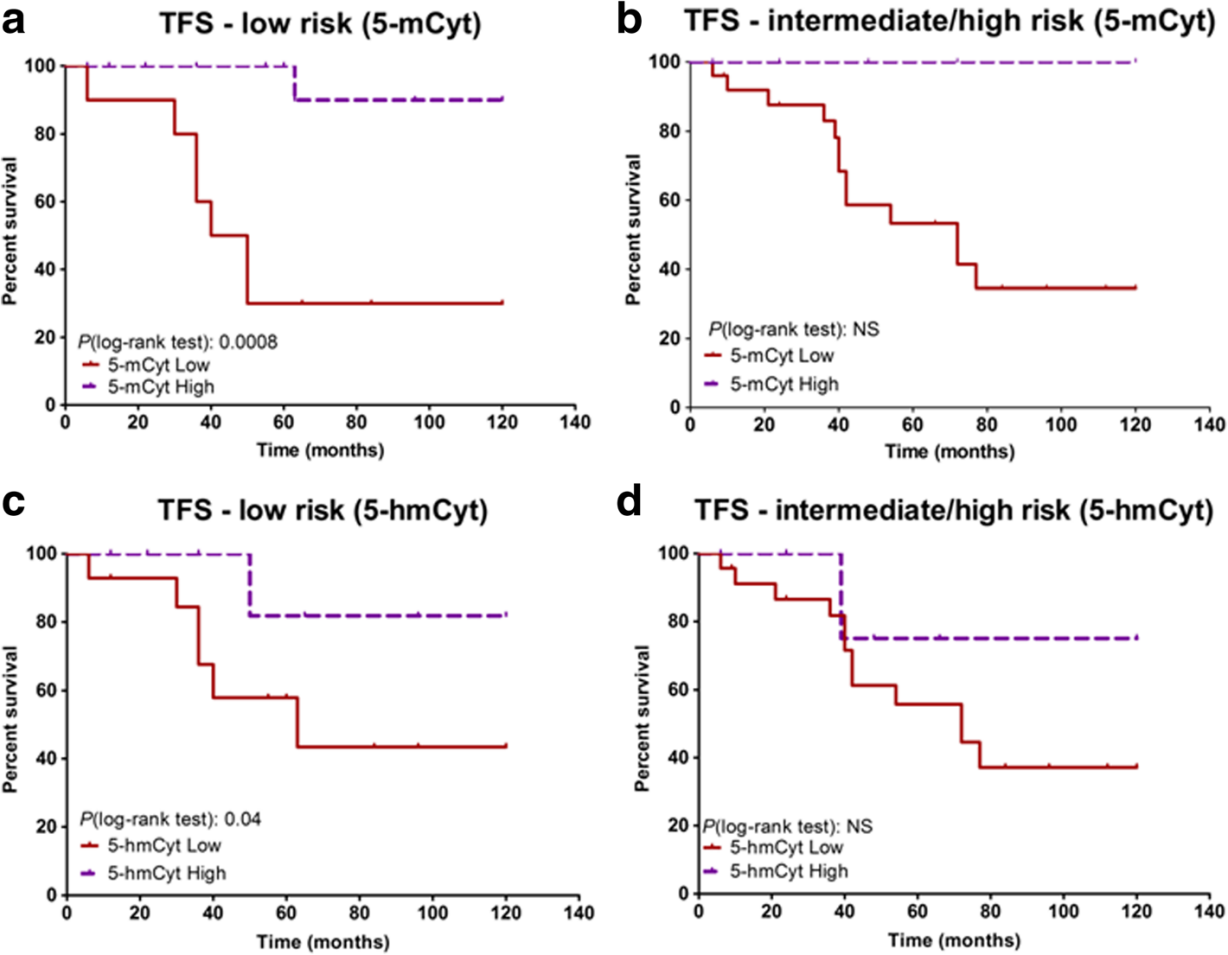
Treatment-free survival (TFS) in a bivariate analysis of cytogenetic and epigenetic characteristics. Influence of DNA methylation (5-methylcytosine, 5-mCyt) on TFS in **a** low-risk chronic lymphocytic leukemia (CLL) patients with isolated deletion (del)13q (*n* = 30) and in **b** intermediate/high-risk CLL patients (*n* = 30). Influence of active DNA demethylation (5-hydroxymethylcytosine, 5-hmCyt) on TFS in **c** low-risk CLL patients with isolated deletion (del)13q and in **d** intermediate/high-risk CLL patients. The Cox regression model of TFS for 5-mCyt and 5-hmCyt was used to identify the optimal cutoff level in order to dichotomize CLL patients into high versus low subgroups. Statistical differences between the Kaplan–Meier curves were calculated using the log-rank test. NS not significant

As depicted in Fig. 3a and presented in Table 2, the Kaplan–Meier survival curves reveal that the 5-mCyt low subgroup had markedly reduced PFS and TFS when considering the CLL patients presenting an isolated del(13q) (PFS 44.5 versus > 120 months, *p* = 0.01, and TFS 45 versus > 120 months, *p* = 0.0008). For 5-hmCyt (Fig. 3c and Table 2), differences were also significant when considering patients with del(13q) as a sole cytogenetic abnormality (PFS 56 versus > 120 months, *p* = 0.02; TFS 63 versus > 120 months, *p* = 0.04). Regarding the cytogenetic intermediate/high-risk group (Fig. 3b, d and Table 3), the same trend was observed for both 5-mCyt and 5-hmCyt but did not reach significance. We conclude from these experiments that combining cytogenetic and epigenetic parameters improves CLL patient outcome prediction and that 5-mCyt levels have a higher impact predominantly on those patients with isolated del(13q).

**Table 2:** Characteristics of the cytogenetic low-risk group according to DNA methylation/hydroxymethylation levels

	CLL patients with del(13q)					
	5-mCyt low (*n* = 12)	5-mCyt high (*n* = 18)	Statistics (*p*)	5-hmCyt low (*n* = 15)	5-hmCyt high (*n* = 15)	Statistics (*p*)
Age at diagnosis, mean (±SD)	65.8 (± 8.5)	61.4 (± 8.0)	NS	66.2 (± 8.6)	60.2 (± 7.2)	NS
Age at study entry, mean (±SD)	69.8 (± 7.6)	67.2 (± 8.4)	NS	69.6 (± 8.4)	66.9 (± 7.7)	NS
Binet stage, No. of patients (%)			0.02			NS
A	6/12 (50%)	16/18 (88.9%)		9/15 (60%)	12/15 (80%)	
B/C	6/12 (50%)	2/18 (11.1%)		6/15 (40%)	3/15 (20%)	
Lymphocytosis (Giga/L), mean (±SD)	53.53 (± 38.30)	33.93 (± 19.9)	NS	34.6 (± 28.6)	48.9 (± 30.0)	NS
IGHV status, No. of patients (%)			NS			NS
Unmutated (≥ 98% homology)	1/7 (14.3%)	0/12 (0%)		1/9 (11.1%)	0/10 (0%)	
Mutated (< 98% homology)	6/7 (85.7%)	12/12 (100%)		8/9 (88.9%)	10/10 (100%)	
CD38 > 30%, No. of patients (%)	5/12 (41.7%)	4/16 (25%)	NS	6/15 (40%)	3/13 (23%)	NS
del(13q), > 80% nuclei (%)	7/12 (58.3%)	3/18 (16.7%)	0.02	4/15 (26.7%)	6/15 (40%)	NS
del(13q), biallelic	4/12 (33.3%)	12/18 (66.7%)	NS	6/15 (40%)	10/15 (66.7%)	NS
LDT from diagnosis, median (months)^a^	12	36	NS	24	18	NS
PFS, median (months)^a^	44.5	120	0.01	56	> 120	0.02
TFS, median (months)^a^	45	> 120	0.0008	63	> 120	0.04

Abbreviations: *5-mCyt* 5-methylcytosine, *5-hmCyt* 5-hydroxymethylcytosine, *NS* not significant, *No*. number, *SD* standard deviation, *IGHV* immunoglobulin heavy-chain variable region, *LDT* lymphocyte doubling time, *PFS* progression-free survival, *TFS* treatment-free survival, *del* deletion

^a^Kaplan–Meier survival analysis

**Table 3:** Characteristics of the cytogenetic intermediate/high-risk group according to DNA methylation/hydroxymethylation levels

	CLL patients with cytogenetic intermediate/high risk					
	5-mCyt low (*n* = 20)	5-mCyt high (*n* = 10)	Statistics (*p*)	5-hmCyt low (*n* = 23)	5-hmCyt high (*n* = 7)	Statistics (*p*)
Age at diagnosis, mean (±SD)	65.8 (± 10.3)	65.7 (± 8.0)	NS	69.6 (± 10.5)	71.1 (± 7.8)	NS
Age at study entry, mean (±SD)	70.3 (± 10.9)	69.1 (± 7.7)	NS	69.6 (± 10.9)	71.1 (± 7.8)	NS
Binet stage, No. of patients (%)			NS			NS
A	8/20 (40%)	6/10 (60%)		9/23 (39.1%)	5/7 (71.4%)	
B/C	12/20 (60%)	4/10 (40%)		14/23 (60.9%)	2/7 (28.6%)	
Lymphocytosis (Giga/L), mean (±SD)	93.5 (± 58.3)	27.9 (± 22.8)	0.0008	83.7 (± 60.0)	32.1 (± 26.5)	0.02
IGHV status, No. of patients (%)			NS			NS
Unmutated (≥ 98% homology)	4/13 (30.8%)	1/5 (20%)		5/15 (33.3%)	0/5 (0%)	
Mutated (< 98% homology)	9/13 (69.2%)	4/5 (80%)		10/15 (66.7%)	5/5 (100%)	
CD38 > 30%, No. of patients (%)	7/18 (38.9%)	4/10 (40%)	NS	7/21 (33.3%)	4/7 (57.1%)	NS
LDT from diagnosis, median (months)^a^	12	24	0.04	16.5	30	NS
PFS, median (months)^a^	40	46	NS	40	83	NS
TFS, median (months)^a^	72	> 120	NS	72	> 120	NS

Abbreviations: *5-mCyt* 5-methylcytosine, *5-hmCyt*, 5-hydroxymethylcytosine, *NS* not significant, *No.* number, *SD* standard deviation, *IGHV* immunoglobulin heavy-chain variable region, *LDT* lymphocyte doubling time, *PFS* progression-free survival, *TFS* treatment-free survival, *del* deletion

^a^Kaplan–Meier survival analysis

### DNA methylation/hydroxymethylation low and high subgroups

We further evaluated, in the cytogenetic low-risk group (Table 2), whether a defective global DNA 5-mCyt status or 5-hmCyt status was associated with other established prognostic factors (age, Binet stage, lymphocytosis, LDT, *IGHV* mutational status, CD38 positivity) and with the subclonal heterogeneity of del(13q). Regarding prognostic factors, patients from the 5-mCyt low subgroup, but not those from the 5-hmCyt low subgroup, had a significantly higher proportion of Binet stage B and C disease (*p* = 0.02). Moreover, as previous studies have shown that CLL patients with isolated del(13q) have a more aggressive disease when presenting a high percentage of 13q deleted nuclei (> 80%) or biallelic deletions in the 13q14 locus, we tested whether these factors were associated with the global DNA 5-mCyt and 5-hmCyt status [15–17]. The 5-mCyt low subgroup presented an increased percentage of 13q deleted nuclei (*p* = 0.02). We failed to observe any difference between the two subgroups (5-mCyt or 5-hmCyt low and high) when biallelic deletions were taken into consideration. Altogether, these results suggest that a defective global DNA methylation status contributes to del(13q) clonal selection and in turn impacts disease outcome in CLL patients with isolated del(13q).

Second, the same analysis was conducted in CLL patients from the intermediate/high-risk groups showing an association between 5-mCyt status, lymphocytosis (*p* = 0.0008), and the LDT (*p =* 0.04) (Table 3). No differences were observed regarding the other prognostic factors (age, Binet stage, *IGHV* mutational status, and CD38 positivity) or when the intermediate/high-risk patients were segregated according to the 5-hmCyt status.

### DNA methylation and demethylation regulators

**Fig. 4:**
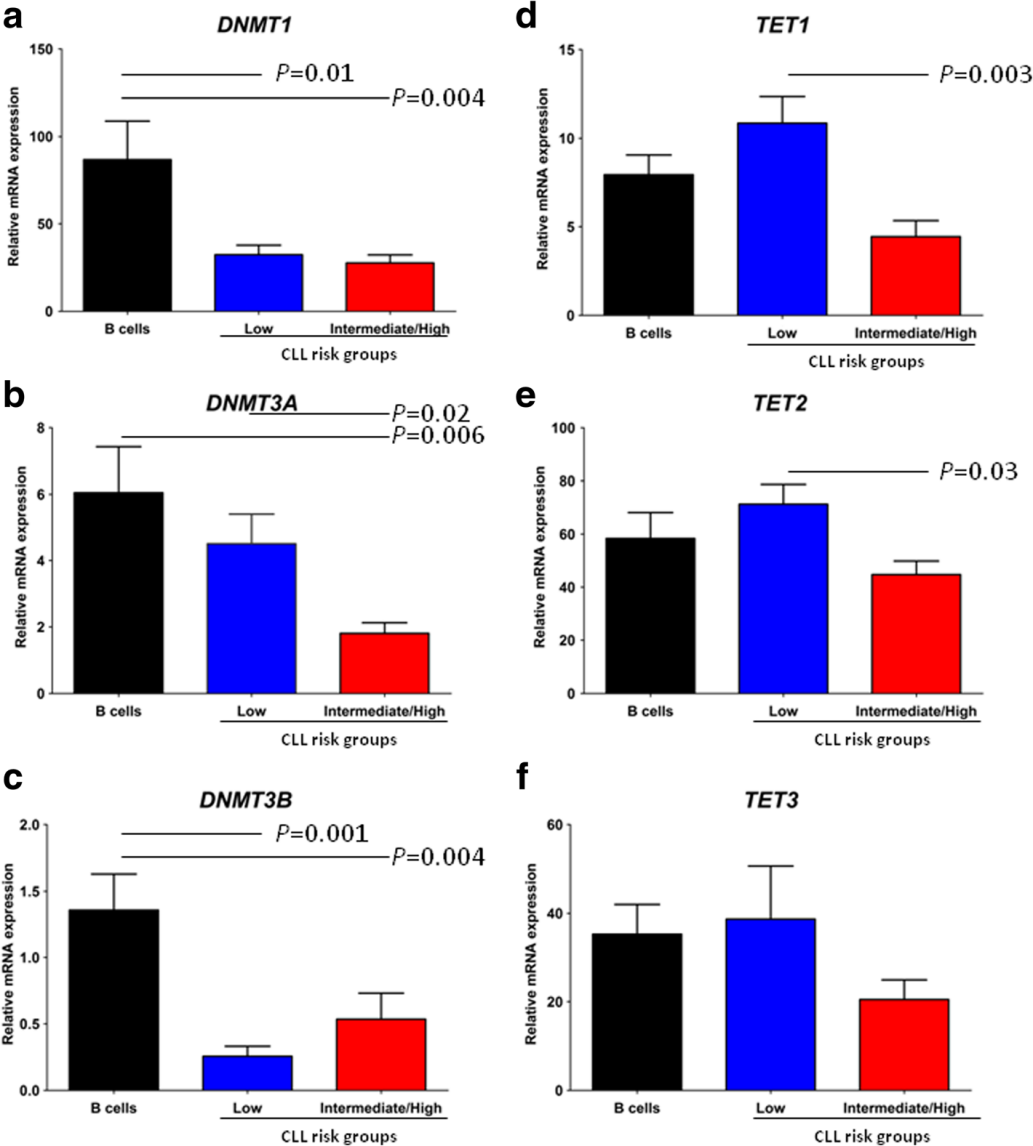
DNA methylation and demethylation regulators according to cytogenetic risk groups **a** *DNMT1*, **b** *DNMT3A*, **c** *DNMT3B*, **d** *TET1*, **e** *TET2*, and **f** *TET3* were tested by real-time quantitative PCR and transcripts expressed relative to *GAPDH*. The mean ± standard error of the mean (SEM) values are indicated, and statistical differences were indicated when Dunn’s correction test was statistically significant (*p* < 0.05)

Finally, transcript quantification of three members of the *DNMT* family (*DNMT1*, *DNMT3A*, and *DNMT3B*) responsible for the methylation of 5-Cyt to 5-mCyt and of the three members of the *TET* family (*TET1*, *TET2*, and *TET3*) responsible for the oxidation of 5-mCyt to 5-hmCyt was performed by RTq-PCR in 11 CLL patients from the low-risk group, 13 CLL patients from the intermediate/high-risk group, and 9 healthy controls (Fig. 4).

Compared to the low-risk group, CLL patients from the intermediate/high-risk group were characterized by a significant reduction of *DNMT3A* (*p* = 0.02), *TET1* (*p* = 0.003), and *TET2* (*p* = 0.03) transcripts. No significant differences were observed for *DNMT1*, *DNMT3B*, and *TET3* transcripts when comparing the CLL intermediate/high-risk group with the CLL low-risk group. However, in opposition to the 5-mCyt results that did not reveal any differences when comparing healthy controls with the low-risk group, reductions were observed in the low-risk group for *DNMT1* (*p* = 0.01) and *DNMT3B* (*p* = 0.001). Concerning the intermediate/high-risk group comparison to healthy controls, differences were observed for *DNMT1* expression (*p* = 0.004), *DNMT3A* (*p* = 0.006), and *DNMT3B* (*p* = 0.04).

## Discussion

Assessment of the cytogenetic status in CLL at diagnosis is routinely performed by using karyotype and FISH analysis for prognostic purposes. However, such an approach poorly integrates clonal evolution, thus indicating that the well-established Dohner’s classification can be improved. Accordingly, we have hypothesized that coupling cytogenetic and epigenetic factors would help predict disease outcome, and in addition, we have confirmed the importance of epigenetic modifications in CLL outcome.

Interplay exists between genetic and epigenetic mechanisms during CLL evolution, and two models were proposed for this co-evolution, one based on a stepwise acquisition model from early clonal mutations/cytogenetic abnormalities (e.g., del(13q)), while in the second model, a simultaneous acquisition was proposed (e.g., del(17p)) [18, 19]. The simultaneous acquisition model is supported by the observation that del(17p) involving the tumor suppressor *TP53* is associated with global DNA demethylation in CLL B cells [10, 20] and by our observation that CLL patients from the cytogenetic intermediate- and/or high-risk groups present a reduction in 5-mCyt and 5-hmCyt levels and in *DNMT3A* and *TET1/2* transcripts. In the second model, as observed in CLL patients with low-risk cytogenetic abnormalities such as del(13q), the global DNA methylation pattern is suspected to be relatively stable over time, and when disease progression occurs, selection of novel global DNA methylation patterns evolves independently of the genetic heterogeneity [18, 21]. Indeed, del(13q) is suspected to be an early event as confirmed by the detection of this cytogenetic abnormality in the CD34^+^ cell population in CLL patients [22]. Our study further supports the stepwise dynamic model of clonal populations in CLL with the del(13q) as an early event which can be associated with epigenetic dysregulations as this clonal population expands. This is highlighted by the observation that CLL patients with a higher percentage (above 80%) of 13q deleted nuclei also have a defective DNA methylation process and a shorter PFS and TFS. Further on, this is also in agreement with the report that CLL patients with a higher percentage of 13q deleted nuclei have a transcriptome pattern similar to del(11p) and del(17q) patients, abnormal miRNA expression, and dysregulated pathways implicated in apoptosis and proliferation (BCR and NF-kappaB signaling) [23]. Altogether, our data and previously published data support a chronology of molecular events involving first the del(13q) and afterwards, as the clone expends, important epigenetic modifications. However, one question remains to be answered which concerns the possible contribution of suppressor genes from the deleted 13q locus to the regulation of the methylation process. Moreover, in this locus, differentially methylated regions have been identified in CLL B cells compared to controls, and they are located within tumor suppressor genes including the deleted in lymphocytic leukemia (*DLEU*)1 and the *DLEU2* variant *ALT1* [20]. Further experiments are required to test whether these suppressor genes are in turn involved in the global DNA methylation process and whether DNA demethylation leads to an adverse clinical outcome per se or through subclonal driver mutations.

*DNMT3A* is thought to be critical in the control of DNA methylation in CLL, and such an assertion is supported by the observation that *DNMT3A* knockdown in rodent hematopoietic stem cells results in CLL development [24] and that *DNMT3A* is present in the top 1% of the under-expressed genes in human CLL B cells [25]. An interaction between the two CLL high-risk factors TCL1 and NOTCH1 with DNMT3A to inhibit its function is also reported in CLL B cells [26, 27]. Accordingly, we have investigated *DNMT*s in CLL B cells and observed that *DNMT1* and *DNMT3B* are downregulated in the cytogenetic low- and intermediate/high-risk groups while *DNMT3A* reduction was restricted to CLLs carrying cytogenetic intermediate/high-risk factors. This result differs from previous publications, which have compared CLL B cells with peripheral blood normal B cells to show, in one case, normal *DNMT1* expression, an upregulation of *DNMT3A*, and a reduction of *DNMT3B* in CLL B cells [28], and in the other case, researchers have reported a reduction in *DNMT1* [10]. These discrepancies could be most likely due to the selection of the control cells as important variations in DNMTs are reported during B cell ontogeny and to the reference gene used for correction [29–31].

Despite its stability, 5-mCyt can be reversed firstly during DNA replication in a passive way or, secondly, by TET proteins which can mediate 5-mCyt oxidation to 5-hmCyt, and in a less efficient fashion to 5-formylcytosine (5-fCyt), and 5-carboxylcytosine (5-CaCyt). Next, transformation to an unmodified Cyt requires the coupled action of thymine DNA glycosylase (TDG) and the base excision repair (BER) system. *TET1* and double *TET1/TET2* knockdown promote B cell malignancies [32, 33]. First reported in myeloid lineage malignant diseases, 5-hmCyt level reduction was related to mutations in *TET*s or their regulators (e.g., *IDH1/2*, *WT1*) [34]. In CLL, DNA demethylation is observed, but mutations in *TET*s and *IDH*s are rare events. However, a reduction in *TET1/3* and *IDH2* was shown to be associated with a poor prognosis [12]. In this report, we could confirm that patients with low mRNA expression of *TET1* and *TET2* were predominantly classified into the cytogenetic intermediate/high-risk group, which is in agreement with our observation that both 5-mCyt and 5-hmCyt levels were reduced in the intermediate- and/or high-risk groups. Van Damme et al. observed a decreased expression of *TET1* in CLL B cells but did not observe an association with DNA hydroxymethylation, and this may be explained, in part, by the small number of patients studied for hydroxymethylation levels [12]. In another study comparing CLL B cells to peripheral blood B cells, *TET2* was reported to be overexpressed and such overexpression was not associated with other clinical and biological markers [35]. The list of the mechanisms controlling *TET* expression in leukemic cells is growing and involves microenvironmental factors [12], hypoxia, reactive oxygen species (ROS), and miRNAs such as miR-15a and miR-29 [36]. Additional experiments are required to better understand the mechanisms controlling active DNA demethylation in CLL and their consequences.

Relevant limitations of our study include the following: (i) global 5-mCyt and 5-hmCyt analysis is likely to reflect variations of repetitive elements (half of the genome) than gene regulatory sequence, which may render comparison with EWAS studies difficult; (ii) the absence of a well-established control B cell for CLL B cells, making difficult the comparison of the transcriptional analysis between normal and tumor cells; and (iii) a transcriptional analysis for *DNMT*s and *TET*s, which does not test enzyme activity.

In conclusion, our study established that loss in the 5-Cyt derivatives, 5-mCyt and, to a lesser extent, 5-hmCyt, during disease progression, better predict the CLL patients’ outcome when associated with the classical cytogenetic analysis. Future work is required to develop routine assays for monitoring DNA methylation during disease course and to test their utility as therapeutic response predictors.

## Materials and methods

### Cells and sample preparation

Clinical and biological data were retrospectively obtained for 127 untreated patients diagnosed with CLL according to the World Health Organization (WHO) classification [37], and samples were collected for 60 CLL patients and 15 healthy volunteers at the Brest University Hospital. Consent was obtained from all individuals and the protocol approved by the Ethical Board at the Brest University Hospital (OFICE, November 26, 2015; ClinicalTrials.gov: NCT03294980; CRB Brest, collection 2008-214), in accordance with the Declaration of Helsinki. Lymphocytes were isolated from peripheral blood mononuclear cells (PBMC) by Ficoll-Hypaque density gradient centrifugation (Eurobio, Courtaboeuf, France), and B cells were further enriched using the Pan B cell Isolation Kit (Miltenyi Biotec GmbH, Bergisch Gladbach, Germany). Cell purity was assessed by fluorescence-activated cell sorting (FACS) analysis and was over 95% for B cells (CD19+). Fluorescence-activated cell sorting (FACS) antibodies (Abs) used were purchased from Beckman Coulter.

### DNA sample preparation and global DNA 5-mCyt and 5-hmCyt level assessment by ELISA.

DNA was extracted from purified B cells using Biosprint 15 DNA Blood Kit (Qiagen, Hilden, Germany). Next, DNA was quantified and its purity assessed using the NanoDrop 2000 Spectrophotometer (Thermo Fisher Scientific, Waltham, MA). An ELISA previously developed in the laboratory was used and adapted to measure global 5-mCyt and 5-hmCyt [38]. Briefly, high affinity microplates (Thermo 269620, Thermo Fisher Scientific) were pre-coated 90 min at room temperature (RT) with 100 μl poly-l-lysine 0.01% (Sigma-Aldrich, St. Louis, MI) to attach DNA. Next, DNA samples adjusted at 2 ng/μl in carbonate/bicarbonate buffer 0.1 M pH 9.6 were denatured at 95 °C for 6 min, kept on ice 5–10 min, and then 100 μl dispensed in each well, in duplicates. The plates were next incubated overnight at 4 °C, three washes with phosphate-buffered saline (PBS)-Tween 0.01% were performed, and 200 μl of PBS with bovine serum albumin (BSA) 1% was added in each well as blocking solution. After 1-h incubation at RT and extensive washing, 100 μl of mouse IgG anti-5-mCyt (diluted 1:5000 in PBS-BSA 1%) or mouse IgG anti-5-hmCyt (1:1000) was added and plates were incubated 2 h at RT. Anti-cytosine derivative Abs were purchased from Abcam (Cambridge, UK). After six washes, 100 μl of alkaline phosphate-labeled goat anti-mouse (Jackson Laboratory, Bar Harbor, ME), diluted at 1:5000 in PBS-BSA 1%, was added and the plate was incubated for 1 h at RT. After three washes, color was developed with 100 μl p-nitrophenyl-phosphate (Sigma-Aldrich) diluted in carbonate/bicarbonate buffer 0.1 M pH 9.6. Plates were kept at 37 °C for 4 h, and optical density (OD) determined at 405 nm using a Titertek Multiscan microplate reader (Flow laboratories, Rockville, MD). Each sample was tested in duplicate, and non-specific background OD (duplicate wells without DNA) was subtracted from the corresponding test sample. For normalization, a reference sample (salmon sperm DNA—Sigma-Aldrich) was included on each plate and indexes calculated using the ratio between the patient OD and the reference sample OD at 200 ng/well.

### Classic cytogenetic and FISH analysis

As previously described [39], cytogenetic analysis was performed on bone marrow or peripheral blood cells cultured for 72 h with B cell mitogens (DSP30+ interleukin-2). Chromosomes were R-banded and the karyotype described according to the International System for Human Cytogenomic Nomenclature (ISCN) [40]. Metaphase and interphase FISH using the Vysis CLL FISH Probe Kit (Abbott, Rungis, France) was performed for the patients analyzed before the year 2012 and then using P53/ATM probe combination (Cytocell, Cambridge, UK) and XL DLEU/LAMP/12cen probe (Metasystems, Altlussheim, Germany). In all the CLL cases, at least 200 interphase nuclei were counted. Positive cases were defined as having ≥ 5% of nuclei with the investigated anomaly. The patients with del(13q) were also separated into monoallelic and biallelic subgroups. Any patient with two clones, harboring both monoallelic and biallelic deletions, were classified into the biallelic del(13q) subgroup.

### Mutational status of *IGHV*

According to the BIOMED-2 consortium guidelines [41], the *IGHV* gene mutation status was determined by sequencing after conducting a PCR multiplex amplification. Briefly, for multiplex PCR, 100 ng of genomic DNA, 0.25 μl of Ampli Taq Gold DNA Polymerase (Applied Biosystems, Foster City, CA), 10 pmol of each primer, 0.2 mM dNTP Mix, 1.5 mM MgCl2, and 1× PCR Buffer II were adjusted to 50 μl with DNase/RNase-free ultrapure distilled water. Next, PCR products were visualized on 2% agarose gel and purified with ExoSAP-IT PCR product cleanup kit (Affymetrix, High Wycombe, UK). Finally, amplicons were sequenced with a Big Dye Terminator v3.1 cycle sequencing kit (Applied Biosystems). Results were analyzed with the database IMGT/HighV-Quest (The international ImMunoGeneTics information system, Montpellier) and a homology sequence > 98% defined an UM status [42].

### RNA sample preparation, reverse transcription, and RTq-PCR

RNA was extracted from purified B cells using the RNeasy Plus Micro Kit (Qiagen). Quantification and purity were assessed using the NanoDrop 2000 Spectrophotometer. Next, RNA (300 ng) was reverse transcribed into cDNA using the Super Script III enzyme and random primers (Invitrogen Life Sciences, Carlsbad, CA). RTq-PCR was carried out in 20 μl mixtures containing 6 μl of template cDNA diluted 1/12 with DNase/RNase-free ultrapure distilled water, 1× Power SYBR® Green PCR Master Mix (Applied Biosystems), and 250 nM of each primer (Table 4) using Applied Biosystems® QuantStudio™ 7 Flex Real-Time PCR System. The PCR conditions were the same for all genes. All assays included a negative control, which consisted of the reaction mixture with no template. Comparison of cycle thresholds was completed with the 2^ΔΔCT^ method using *GAPDH* as an endogenous control.

**Table 4:** Primers used for real-time quantitative PCR

Symbol	Gene description	Forward primer	Reverse primer
*GAPDH*	Glyceraldehyde-3-phosphate dehydrogenase	TGCCCTCAACGACCACTTT	GGTCCAGGGGTCTTACTCCTT
*DNMT1*	DNA methyltransferase 1	CCTGTACCGAGTTGGTGATGGT	CCTTCCGTGGGCGTTTC
*DNMT3A*	DNA methyltransferase 3 alpha	CTCCTGTGGGAGCCTCAATGTTACC	CAGTTCTTGCAGTTTTGGCACATTCC
*DNMT3B*	DNA methyltransferase 3 beta	ACCACCTGCTGAATTACTCACGC	GATGGCATCAATCATCACTGGATT
*TET1*	tet methylcytosine dioxygenase 1	AATGGAAGCACTGTGGTTTG	ACATGGAGCTGCTCATCTTG
*TET2*	tet methylcytosine dioxygenase 2	AATGGCAGCACATTGGTATG	AGCTTCCACACTCCCAAACT
*TET3*	tet methylcytosine dioxygenase 3	TTGCGTCGAACAAATAGTGG	CCCGTGTAGATGACCTTCTC

### Clinical outcome endpoints

PFS was defined as the time from disease discovery to disease progression. Disease progression was considered either as the shift from Binet stage A to Binet stage B/C or as a short LDT of less than 6 months. TFS was defined as the interval between the date of disease discovery and the date of treatment initiation.

### Statistical analysis

The profile likelihood method using a Cox regression model of TFS was used in univariate analysis to determine the optimal threshold and stratify patients into two groups. This analysis was computed using the Survival and SurvMisc R packages [43]. LDT, TFS, and PFS analyses were next performed using Kaplan–Meier curves, and prognosis differences between groups were assessed with a log-rank test. Continuous data are described as mean ± standard error of the mean (SEM). Differences among groups were analyzed by the Kruskall–Wallis test and the Dunn test was used for post hoc comparisons, or the Fisher exact test for categorical data. Following normality and equality of variance tests, nominal values were compared to controls using the student *t* test or alternatively by using a nonparametric test (Mann–Whitney rank sum test). *p* values under 0.05 were considered significant. Statistical analyses were performed using GraphPad Prism 7.0 (La Jolla, CA).
